# Immature Skeletal Myotubes Are an Effective Source for Improving the Terminal Differentiation of Skeletal Muscle

**DOI:** 10.3390/cells13242136

**Published:** 2024-12-23

**Authors:** Seung Yeon Jeong, Jun Hee Choi, Paul D. Allen, Eun Hui Lee

**Affiliations:** 1 Department of Physiology, College of Medicine, The Catholic University of Korea, Seoul 06591, Republic of Korea; tjdus0560@catholic.ac.kr (S.Y.J.); junhee@catholic.ac.kr (J.H.C.); 2 Department of Medical Sciences, Graduate School, The Catholic University of Korea, Seoul 06591, Republic of Korea; 3 Department of Anesthesiology, Graduate School of Medicine, University of Tennessee, Knoxville, TN 37920, USA; paul_allen@hms.harvard.edu

**Keywords:** immature myotube, co-differentiation, terminal differentiation

## Abstract

Injured or atrophied adult skeletal muscles are regenerated through terminal differentiation of satellite cells to form multinucleated muscle fibers. Transplantation of satellite cells or cultured myoblasts has been used to improve skeletal muscle regeneration. Some of the limitations observed result from the limited number of available satellite cells that can be harvested and the efficiency of fusion of cultured myoblasts with mature muscle fibers (i.e., terminal differentiation) upon transplantation. However, the possible use of immature myotubes in the middle of the terminal differentiation process instead of satellite cells or cultured myoblasts has not been thoroughly investigated. Herein, myoblasts (Mb) or immature myotubes on differentiation day 2 (D2 immature myotubes) or 3 (D3 immature myotubes) were transferred to plates containing D2 or D3 immature myotubes as host cells. The transferred Mb/immature myotubes on the plates were further co-differentiated with host immature myotubes into mature myotubes in six conditions: Mb-to-D2, D2-to-D2, D3-to-D2, Mb-to-D3, D2-to-D3, and D3-to-D3. Among these six co-differentiation conditions, the D2-to-D3 co-differentiation condition exhibited the most characteristic myotube appearance and the greatest availability of Ca^2+^ for skeletal muscle contraction. Compared with non-co-differentiated control myotubes, D2-to-D3 co-differentiated myotubes presented increased MyoD and myosin heavy chain II (MyHC II) expression and increased myotube width, accompanied by parallel and swirling alignment. These increases correlated with functional increases in both electrically induced intracellular Ca^2+^ release and extracellular Ca^2+^ entry due to the increased expression of ryanodine receptor 1 (RyR1), sarcoplasmic/endoplasmic reticulum Ca^2+^-ATPase 1a (SERCA1a), and stromal interaction molecule 1 (STIM1). These increases were not detected in any of the other co-differentiation conditions. These results suggest that in vitro-cultured D2-to-D3 co-differentiated mature myotubes could be a good alternative source of satellite cells or cultured myoblasts for skeletal muscle regeneration.

## 1. Introduction

Skeletal muscle is composed of muscle fibers that are long multinucleated cells which are mimicked in culture by ‘differentiated myotubes’ [1]. Compared with other tissues or organs, adult skeletal muscle undergoes a remarkable process, referred to as regeneration through terminal differentiation [2]. For terminal differentiation, quiescent satellite cells can be activated by various cues, and the activated satellite cells proliferate (called ‘myoblasts’), fuse with existing muscle fibers, and form longer and/or thicker multinucleated myotubes. Skeletal muscle injury is one of the primary cues for this process, and the consequent terminal differentiation allows the regeneration of skeletal muscle at the injury site [3]. Quiescent satellite cells in skeletal muscle are located between the basement membrane and the plasma membrane of muscle fibers [4]. In myoblasts, the expression of two differentiation/myogenic factors, namely MyoD and myogenin, is suppressed by c-Myc [5]. The release of the repression of MyoD and myogenin prevents further proliferation of myoblasts and, instead, induces the terminal differentiation of myoblasts into myotubes [6].

Skeletal muscle contraction occurs via excitation–contraction (EC) coupling [7,8]. In brief, the nerve firing in the neuromuscular junction is transmitted to the slow voltage-gated Ca^2+^ channels (dihydropyridine receptors, DHPRs) in the transverse tubule (t-tubule) membrane causing a conformational change in the II-III loop of the activated DHPR which then activates the calcium release channel (type 1 ryanodine receptor, RyR1) in the sarcoplasmic reticulum (SR) membrane via physical interactions. This event results in the release of intracellular Ca^2+^ from the SR into the cytosol. The increase in cytosolic Ca^2+^ allows the interaction of the contractile actin and myosin filaments, inducing skeletal muscle contraction. In addition to the release of intracellular Ca^2+^ from the SR into the cytosol, store-operated extracellular Ca^2+^ entry (SOCE) into the cytosol helps to maintain skeletal muscle contraction [9]. SOCE is mediated mainly by Orai1 (a Ca^2+^ entry channel in the t-tubule/plasma membrane) and stromal interaction molecule 1 (STIM1, a Ca^2+^ sensor in the SR membrane). In addition to Orai1, canonical transient receptor potential cation channels (TRPCs) also function as SOCE-mediating Ca^2+^ channels in skeletal myotubes [10]. Sarcoplasmic/endoplasmic reticulum Ca^2+^-ATPase (SERCA, a Ca^2+^ pump on the SR membrane) causes the relaxation of skeletal muscle by pumping cytosolic Ca^2+^ back into the SR [7,11]. Timely and adequate expression of EC coupling- or SOCE-mediating proteins during the progression of terminal differentiation is required for accurate regulation of the increase and decrease in the cytosolic Ca^2+^ levels during skeletal muscle contraction and relaxation [2,12].

Loss of skeletal muscle due to a disease or process where the degradation of skeletal muscle proteins exceeds their synthesis induces sarcopenia, leading to atrophy [13]. Skeletal muscle atrophy is induced by malnutrition, resulting from causes such as food deprivation or cancer cachexia, disuse or decreased use of skeletal muscle due to immobilization of a limb or accidental denervation, skeletal muscle diseases, systemic diseases, or a sedentary lifestyle, or the treatment of other diseases, such as cancer [14]. In addition to muscle loss caused by diseases, loss of skeletal muscle also occurs during the normal aging process. Partial muscle atrophy and sarcopenia are accompanied by muscle weakness, manifested as decreased force production and decreased fatigue resistance.

The transplantation of freshly isolated satellite cells or cultured primary myoblasts into injured muscles or muscles with disease-associated atrophy has been used to improve skeletal muscle regeneration and function by repopulating myogenic cells at the sites of injury or atrophy [15,16]. However, the number of isolated satellite cells that can be harvested is limited due to the small number of satellite cells that remain activatable in adult muscle [15]. Cultured primary myoblasts have been used much more than satellite cells in studies of cell transplantation; however, they are much less efficient for terminal differentiation than freshly isolated satellite cells [17].

Considering the limitations of isolated satellite cells or cultured primary myoblasts [15,17] and the properties of terminal differentiation showing serial fusion of myoblasts to form long multinucleated mature myotubes [2], we questioned whether the in vitro co-differentiation of immature myotubes at different stages of terminal differentiation to mature myotubes could provide the benefit of overcome these limitations. Here, we evaluated co-differentiated myotube morphology and Ca^2+^ mobilization as functional properties of co-differentiated myotubes.

## 2. Materials and Methods

### 2.1. Ethical Approval

The methods were carried out in accordance with the regulations and guidelines of the College of Medicine at The Catholic University of Korea (Ethics Approval Code: 2017-0117-01, 17 January 2017). The conditions of the sites where the animal work and surgical interventions were conducted and the procedures for all surgical interventions were in accordance with the Laboratory Animals Welfare Act, the Guide for Care and Use of Laboratory Animals, and the Guidelines and Policies for Rodent Survival Surgery, and were approved by the Institutional Animal Care and Use Committee (IACUC) of the College of Medicine at The Catholic University of Korea. All protocols for the experiments were approved by the IACUC of the College of Medicine at The Catholic University of Korea.

### 2.2. Cell Culture and Co-Differentiation

**Figure 1:**
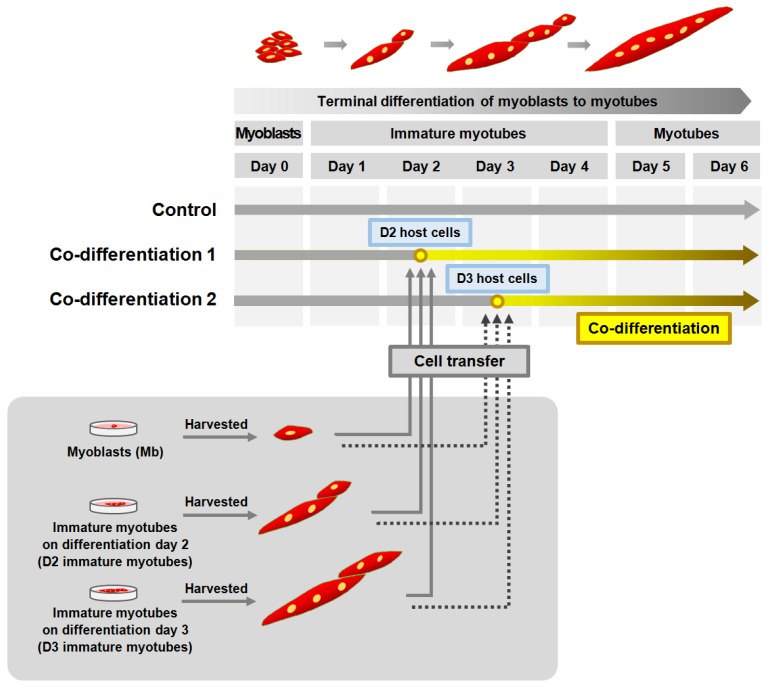
Schematic diagram of the co-differentiation conditions. Day 0 to day 6: differentiation days 0 to 6 during the terminal differentiation of myoblasts into myotubes. Myotubes: fully differentiated multinucleated cells on differentiation day 5 or 6. D2 and D3 host cells: immature myotubes on differentiation day 2 and 3, respectively. Co-differentiation 1 and co-differentiation 2: conditions with D2 and D3 myotubes, respectively, as host cells.

Primary skeletal myoblasts were derived from the skeletal muscle of mouse hind limbs (two 14-day-old males and one female C57BL/6 fetus) via a single-cell cloning method [18]. Myoblasts used in this study were all the progeny proliferated from a single myoblast. Cell culturing was carried out as previously described [19]. Briefly, the isolated primary myoblasts were expanded on collagen-coated culture plates in growth medium (F10 nutrient mixture containing 20% FBS, 100 U/mL penicillin, 100 μg/mL streptomycin, 2 mM L-glutamine, and 20 nM basic fibroblast growth factor) at 37 °C in a 5% CO_2_ incubator. For the differentiation of myoblasts into myotubes, myoblasts were seeded in 10 cm-diameter dishes (for the preparation of myotube lysates, observation of myotubes, or counting of myoblasts), 6-well plates (for measurement of myotube width), or 96-well plates (for single-cell Ca^2+^ imaging experiments) coated with Matrigel (Corning, Glendale, AZ). When the myoblasts were approximately 65% confluent, the growth medium was replaced with differentiation medium (5% heat-inactivated horse serum and low-glucose DMEM without added growth factors), and the plates were placed in an 10% CO_2_ incubator (37 °C) to induce terminal differentiation. For the co-differentiations, myoblasts prior to differentiation or immature myotubes on differentiation day 2 or 3 (Mb, D2 immature myotubes, and D3 immature myotubes, respectively; Figure 1) were transferred to culture dishes or plates containing either D2 or D3 immature myotubes and co-differentiated into mature myotubes until day 5 or 6 on the basis of the differentiation timeline of the host cells (Figure 1). To examine the appearance of cells, myoblasts, immature myotubes, and co-differentiated myotubes were evaluated by microscopy at the same locations on the host plates on differentiation days 0, 3, 4, 5, and 6. All reagents used for the cell cultures were obtained from Invitrogen (Waltham, MA, USA). Please check Table S1 for the definitions of different types of muscle cells mentioned in this study.

### 2.3. Preparation of the Transferred Cells and Cell Transfer Procedures

To prepare the transferred cells (myoblasts, D2 immature myotubes, or D3 immature myotubes), after the removal of their culture media, the cells were harvested from the culture dishes or plates by incubating them with 0.25% trypsin (Thermo Fisher Scientific, Waltham, MA, USA) for 2 min. The cells were pelleted using a centrifuge (100 rcf for 5 min, acceleration at level 3, and free deceleration, Eppendorf 5810R, Enfield, CT, USA) and the trypsin-containing supernatant was removed by suction. The cell pellet was then gently suspended in 1 mL of host cell media and transferred to the host cell culture dishes.

### 2.4. Counting of Myoblasts and Width Measurement of Myotubes

**Figure 2:**
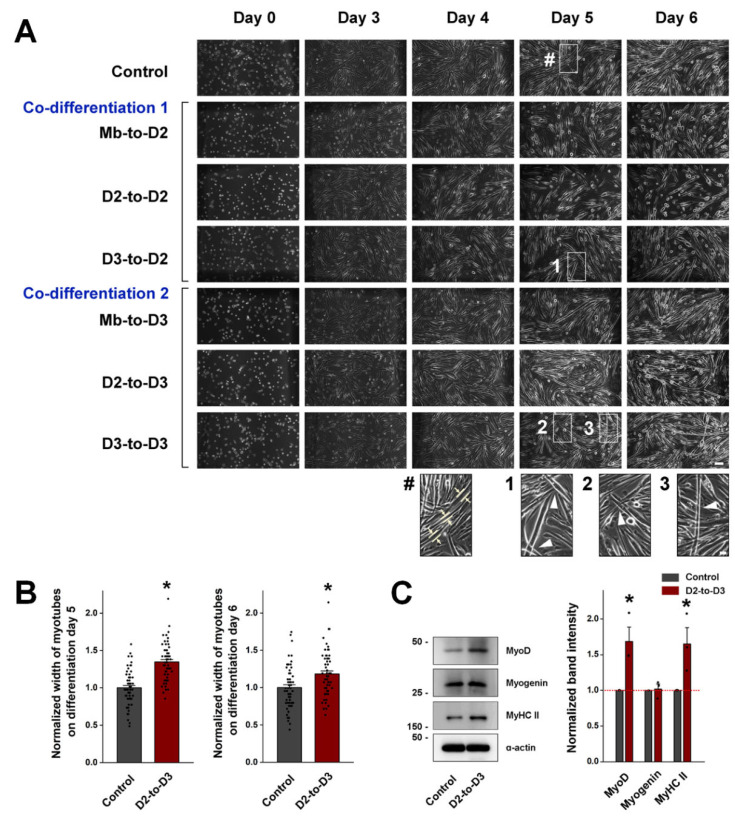
Appearance of co-differentiated myotubes, myotube widths, and expression levels of myogenic factors in D2-to-D3 co-differentiated myotubes. (**A**) Cells on differentiation day 0 (myoblasts) and co-differentiated myotubes on differentiation days 3, 4, 5, and 6 were observed under a microscope. Day 0, day 3, day 4, day 5, and day 6: differentiation days 0, 3, 4, 5, and 6, respectively, during terminal differentiation. Enlarged images of the areas that were indicated by # or numbers (1 to 3) are shown at the bottom. Light yellow arrows in the first enlarged image (marked by #) indicate the thickest part of the myotubes, showing how we measured myotube width. Intersecting myotubes are indicated by white arrowheads in the enlarged images 1 to 3. The main figure can be downloaded and zoomed-in with clear and good quality (Figure S2). The white bar in the bottom right-hand corner of the main figure represents 100 µm. The white bar in the bottom right-hand corner of the enlarged image 3 represents 25 µm. (**B**) The width of D2-to-D3 co-differentiated myotubes on differentiation days 5 and 6 was measured. Each value was normalized to the mean value in the control group, and the data are summarized as histograms (mean ± SE for 60 myotubes from 60 different spots in 5 different wells per condition, Table S5). The mean values are presented in Table 1. (**C**) Lysates of D2-to-D3 co-differentiated myotubes on differentiation day 5 were subjected to immunoblot analysis with antibodies against MyoD, myogenin, and MyHC II. α-actin was used as the loading control. A representative result is presented. The expression level of each protein was normalized to the mean value in the control group, and the data are summarized as histograms (mean ± SE of three independent experiments, Table 2). * Significant difference compared with the control group (*p* < 0.05).

To count the cells on differentiation day 0 (i.e., myoblasts), myoblasts on 10-cm dishes were visualized with an inverted microscope (Nikon Eclipse TS100, Nikon Instruments, Inc., Melville, NY, USA) equipped with a monochrome camera (ProgRes MF, JENOPTIK Optical Systems, Inc., Jena, Germany) [19,20]. Fields of view (1000 μm wide and 600 μm long) from different dishes were randomly selected via PregRes Capture Pro (v2.8.8, JENOPTIK Optical Systems, Inc.), and the myoblasts in each field of view were counted (Tables S2 and S3). To measure the width of myotubes, myotubes in 6-well plates were visualized with the same microscope on differentiation days 5 and 6 in a 1250 μm wide and 950 μm long unit area. The width of the thickest part of branchless myotubes were measured using ImageJ (https://imagej.net), as indicated by light yellow arrows in Figure 2A. The average width of the myotubes was calculated.

### 2.5. Immunoblot Analysis

**Figure 3:**
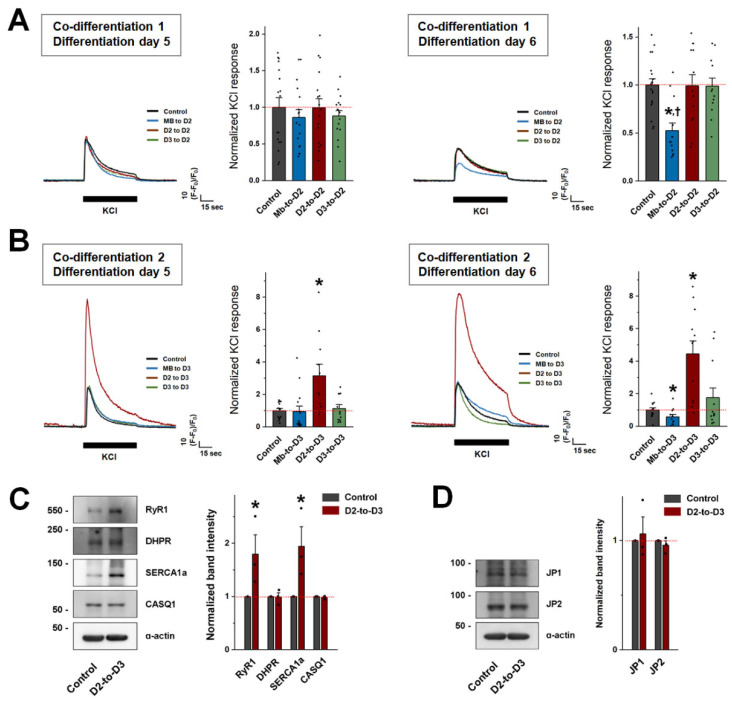
Intracellular Ca^2+^ release for skeletal muscle contraction in co-differentiated myotubes and the expression levels of EC coupling-mediating proteins in D2-to-D3 co-differentiated myotubes. KCl (60 mM) was applied to co-differentiated myotubes on differentiation days 5 (left) and 6 (right) for 1 min under co-differentiation conditions 1 (**A**) and 2 (**B**), and intracellular Ca^2+^ release from the SR to the cytosol through RyR1 in response to KCl was measured. A representative trace for each group is shown. The histograms show the peak amplitude normalized to the mean value in the control group. The results are presented as the mean ± SE for the number of myotubes shown in parentheses in Table 3. Lysates of D2-to-D3 co-differentiated myotubes on differentiation day 5 were subjected to immunoblot analysis with antibodies against major EC coupling-mediating proteins (**C**) and triad formation-mediating proteins (**D**). α-actin was used as the loading control. Three independent experiments per protein were conducted. A representative result is presented. The histograms show the expression level of each protein normalized to the mean value in the control group (mean ± SE of three independent experiments, Table 2). * Significant difference compared with the control group (*p* < 0.05). ^†^ Significant difference compared with the corresponding value on differentiation day 5 (*p* < 0.05). CASQ1, calsequestrin 1.

For the immunoblot analysis, D2-to-D3 co-differentiated myotubes on differentiation day 5 were harvested from 10 cm plates and lysed in 150–200 μL of lysis buffer (1% Triton X-100, 10 mM Tris/HCl (pH 7.4), 1 mM Na_3_VO_4_, 10% glycerol, 150 mM NaCl, 5 mM EDTA, and protease inhibitor cocktail tablets) per 10 cm dish overnight at 4 °C with gentle mixing. Then, 5–10 μg of total protein from the myotube lysates (depending on the protein of interest) was separated by electrophoresis on a 10% or 12% sodium dodecyl sulfate–polyacrylamide gel electrophoresis gel, as previously described [19,20]. Briefly, the proteins in the gel were transferred to a polyvinylidene difluoride membrane at 100 V for 1 h. The membrane was blocked with 5% (wt/vol) nonfat dry milk dissolved in PBS to avoid nonspecific interactions between the antibodies and proteins and incubated for 16 h at 4 °C with the corresponding primary antibody. After incubation, the membrane was washed three times with PBS containing 0.1% Tween 20 and was then incubated with horseradish peroxidase-conjugated anti-goat, anti-mouse, or anti-rabbit secondary antibodies at room temperature (24 °C) for 45 min. The membrane was then washed three times with PBS containing 0.1% Tween 20, and immunoreactive bands were visualized using a SuperSignal West Pico PLUS chemiluminescent substrate (Pierce, Rockford, IL, USA). Three independent experiments were conducted for each protein, and the representative results are shown in Figure 2C, Figure 3C,D and Figure 5D. The density of each protein band was normalized to the mean value of the control band from the untransferred control myotubes, and the data are summarized as histograms. Information on the antibodies used in this study is listed in Table S4. The specificity of the antibodies used was confirmed in previous reports.

### 2.6. Single-Cell Ca^2+^ Imaging Experiments

Single-cell Ca^2+^ imaging experiments were carried out as previously described [19]. Briefly, co-differentiated myotubes on differentiation day 5 or 6 were loaded with 5 μM Fluo-4 AM (Invitrogen) in imaging buffer (25 mM HEPES, 125 mM NaCl, 5 mM KCl, 2 mM KH_2_PO_4_, 2 mM CaCl_2_, 6 mM glucose, 1.2 mM MgSO_4_, and 0.05% BSA; pH 7.4) for 45 min at 37 °C for all experiments except for the measurement of cytosolic Ca^2+^ levels, which was performed with 5 μM Fura-2 AM (Invitrogen). To measure the cytosolic Ca^2+^ levels of myotubes, Ca^2+^ calibration with Fura-2 AM was conducted with a Calcium Calibration Buffer Kit #1 (Thermo Fisher Scientific). In brief, 10 mM CaEGTA (with 100 mM KCl and 30 mM MOPS (pH 7.2) in deionized water) was mixed with 10 mM K_2_EGTA (with 100 mM KCl and 30 mM MOPS (pH 7.2) in deionized water) to prepare buffers with various [Ca^2+^]_free_ values ranging from 0 to 39 μM. The dissociation constant of Fura-2 was calculated from a curve generated by scanning the excitation (340 nm) or emission of Fura-2 (510 nm) in the presence of 11 different Ca^2+^ concentrations (0.000 μM, 0.017 μM, 0.038 μM, 0.065 μM, 0.100 μM, 0.150 μM, 0.225 μM, 0.351 μM, 0.602 μM, 1.350 μM, and 39.000 μM [Ca^2+^]_free_). Single-cell Ca^2+^ imaging was performed with a high-speed monochromator (FSM150Xe with a 75 W xenon lamp, Bentham Instruments, Reading, Berkshire, UK) and an inverted stage microscope (Nikon Eclipse TS100, Nikon Instruments, Inc.). Caffeine (20 mM) or KCl (60 mM) was dissolved in imaging buffer and applied to the myotubes for 1 min via an automated perfusion system (AutoMate Scientific, Berkeley, CA, USA). For measurements of releasable Ca^2+^ from the SR, thapsigargin (TG; 2.5 mM) in DMSO (<0.05%) was applied to myotubes in the absence of extracellular Ca^2+^ imaging buffer with 5 mM EGTA. For SOCE measurements, Ca^2+^ in the SR was depleted with TG (2.5 mM) in the absence of extracellular Ca^2+^, and 2 mM Ca^2+^ was added to myotubes to measure SOCE. The data were analyzed and visualized with image acquisition and analysis software (High-Speed InCyt Im2 for cytosolic Ca^2+^ levels and Im1 for other data, v5.29; Intracellular Imaging Inc., Cincinnati, OH, USA). To analyze Ca^2+^ mobilization, the peak amplitudes (which showed patterns similar to those of the peak areas) were measured. To measure Ca^2+^ mobilization during SOCE or the amount of Ca^2+^ releasable from the SR, the peak areas were analyzed. Other reagents that were not specifically mentioned but used for single-cell Ca^2+^ imaging were obtained from Sigma-Aldrich (Burlington, MA, USA).

### 2.7. Statistical Analysis

**Figure 4:**
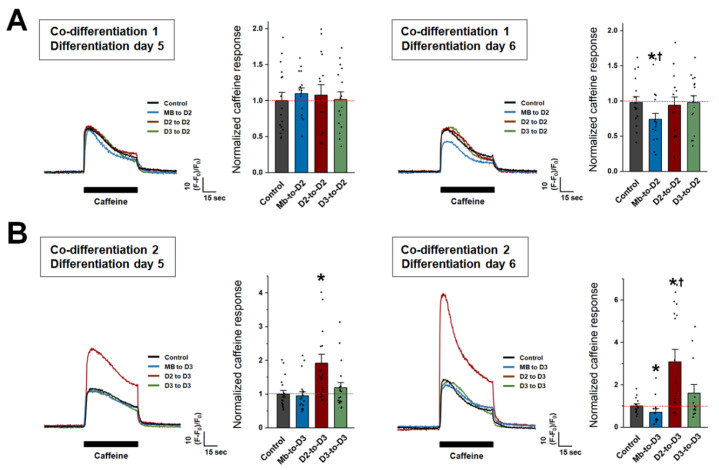
Intracellular Ca^2+^ release through RyR1 in co-differentiated myotubes. Caffeine (20 mM) was applied to co-differentiated myotubes on differentiation days 5 (left) and 6 (right) for 1 min under co-differentiation conditions 1 (**A**) and 2 (**B**), and intracellular Ca^2+^ release from the SR to the cytosol through RyR1 was measured. A representative trace for each group is shown. The histograms show the peak amplitude normalized to the mean value in the control group. The results are presented as the mean ± SE for the number of myotubes shown in parentheses in Table 4. * Significant difference compared with the control group (*p* < 0.05). ^†^ Significant difference compared with the corresponding value on differentiation day 5 (*p* < 0.05).

The results are presented as the mean ± SE for the numbers of myoblasts, myotubes, or experiments indicated in the figure legends and tables. Significant differences (*p* < 0.05) were identified via one-way ANOVA with Tukey’s post hoc test (for the data in Figure 3A,B and Figure 4) or an unpaired *t* test (for other data) (Origin 2019 b, OriginLab Northampton, MA, USA). The graphs were generated with Origin 2019 b.

## 3. Results

### 3.1. Immature Myotubes at Different Stages of Early Terminal Differentiation Were Co-Differentiated to Determine the Benefit of Fusion Between Immature Myotubes

Considering the location of satellite cells and the relatively long distances separating these cells in skeletal muscle fibers, the activation of satellite cells to proliferate, become myoblasts, and then differentiate to regenerate skeletal muscle might not be precisely synchronized. This possibility suggests that, during regeneration via terminal differentiation, both fusion between myoblasts and fusion between immature myotubes in different stages of early terminal differentiation can occur.

To examine the possibility and benefit of fusion between immature myotubes in different stages of terminal differentiation, we transferred myoblasts or immature myotubes (in different stages of terminal differentiation after culture in differentiation medium for 2 days (D2 immature myotubes) or for 3 days (D3 immature myotubes)) from one culture dish to a second culture dish containing D2 or D3 immature myotubes (called host cells in Figure 1), as described in Materials and Methods section. We did not use cells differentiated for only one day or cells cultured in differentiation medium for 4 or 5 days because they either had shapes and characteristics similar to undifferentiated myoblasts or appeared to be fully differentiated mature myotubes with long tubular shapes, respectively. After the transfer of myoblasts, D2 immature myotubes, or D3 immature myotubes to the host cell cultures, the cells were co-differentiated in differentiation medium until day 6, on the basis of the differentiation timeline of the host cells. Under the six different co-differentiation conditions, mature myotubes are referred to as myoblast (Mb)-to-D2, ‘D2-to-D2’, or ‘D3-to-D2’ myotubes when the host cells were D2 immature myotubes (‘co-differentiation 1’ in Figure 1) or as ‘Mb-to-D3’, ‘D2-to-D3’, or ‘D3-to-D3’ myotubes when the host cells were D3 immature myotubes (‘co-differentiation 2’ in Figure 1).

Because myotubes are multinucleated cells with different degrees of fusion, it is meaningless to count the number of immature or mature myotubes. In the control groups without cell transfer, terminal differentiation was initiated when the number of myoblasts per unit viewed (1000 μm wide and 600 μm long) was 137.53 ± 19.96. The confluence of the control myotubes on differentiation day 5 was approximately 90%, which was indistinguishable from those of the co-differentiated myotubes on differentiation day 5 when the numbers of myoblasts presented in Table S2 were used for preparing the transferred cells or host cells. In addition, to examine how many cells were transferred to the host cell plates, attachment rates were measured by transferring cells to empty plates and keeping them in differentiation conditions for 1 h, 3 h, or 7 h (Figure S1). Myoblasts showed an attachment rate of 48.33 ± 4.56% (i.e., approximately 50%) at 1 h after the transfer, which did not significantly change over the time (4 h or 7 h after the transfer). The same tendency of attachment rate was found when D2 or D3 immature myotubes were transferred to the empty plates.

### 3.2. D2-to-D3 Co-Differentiated Myotubes Showed the Best Appearance

To evaluate the appearance of the myotubes, the control and co-differentiated myotubes were observed under a microscope on differentiation days 5 and 6 (Figure 2A). The Mb-to-D2, Mb-to-D3, and D2-to-D2 cultures contained long myotubes with a parallel and swirled alignment, which is a characteristic of well-differentiated myotubes in culture (i.e., long and parallel myotubes with a swirled side-by-side alignment without intersection or entanglement). The appearance of these myotubes was indistinguishable from that of the control myotubes without cell transfer (control group). D2-to-D3 co-differentiated myotubes on differentiation day 5 presented the best parallel and swirled alignment. Interestingly, in the co-differentiation of D3 immature myotubes with either D2 or D3 host cells, the myotubes on differentiation day 5 intersected each other much more frequently (enlarged images 1 to 3 in the D3-to-D2 and D3-to-D3 images in Figure 2A, indicated by white arrowheads).

To evaluate the degree of terminal differentiation, the width of the co-differentiated myotubes was measured on differentiation days 5 and 6, as indicated by light yellow arrows in the enlarged image # in Figure 2A. In all co-differentiations with myoblast transfer, the myotube width was significantly decreased compared with that in the control group on differentiation day 5 but recovered on differentiation day 6, regardless of the host cell differentiation stage (Table 1). Compared with that of the control myotubes, the width of D3-to-D3 co-differentiated myotubes was significantly increased on differentiation day 5, but this increase was no longer observed on differentiation day 6 (Table 1). The width of D2-to-D3 co-differentiated myotubes was significantly greater than that of control myotubes on both differentiation days 5 and 6 (but to a lesser extent on differentiation day 6 than day 5, Figure 2B and Table 1). Neither D2-to-D2 nor D3-to-D2 co-differentiated myotubes showed any change in width. The number of spots observed for the measurement of myotube width is listed in Table S5.

Overall, D2-to-D3 co-differentiated myotubes presented ’the best appearance’ among the six different types of co-differentiated myotubes, i.e., the increase in myotube width and the best parallel and swirled side-by-side alignment of myotubes, as shown in Figure 2A. Considering that there was no significant difference in the number of transferred cells or host cells among the six co-differentiation conditions (Table S2), the best appearance of the D2-to-D3 co-differentiated myotubes was not due to a difference in cell number.

To examine the expression levels of myogenic factors that regulate terminal differentiation, lysates of D2-to-D3 co-differentiated myotubes obtained on differentiation day 5 were subjected to immunoblot analysis of MyoD, myogenin, and MyHC II expression (Figure 2C). The expression of MyoD was significantly greater in D2-to-D3 co-differentiated myotubes than in control myotubes (Table 2), and this increase may have contributed to the increased width of D2-to-D3 co-differentiated myotubes. Consistent with this finding, the expression of MyHC II was also increased (Figure 2C). In addition, D2-to-D3 co-differentiated myotubes on differentiation day 5 also showed a higher fusion index than the control myotubes (70.00 ± 2.11 vs. 54.83 ± 2.90%, Figure S3).

### 3.3. D2-to-D3 Co-Differentiated Myotubes Showed Increased Intracellular Ca^2+^ Release

To examine the functional relevance of the best appearances of the D2-to-D3 co-differentiated myotubes, intracellular Ca^2+^ release was measured in all six types of co-differentiated myotubes on differentiation day 5 after membrane depolarization by KCl. KCl induces concentration-dependent membrane depolarization and, at this concentration, acted similarly to a nerve stimulus to cause the interaction of DHPR and RyR1, causing the Ca^2+^ release from the SR to the cytosol, thus mimicking the Ca^2+^ release from the SR to the cytosol during EC coupling. Mb-to-D2 and Mb-to-D3 co-differentiated myotubes presented similar levels of Ca^2+^ following KCl depolarization (Figure 3A,B, left). However, on differentiation day 6, compared with control myotubes, Mb-to-D2 and Mb-to-D3 co-differentiated myotubes presented significantly decreased responses to KCl (Figure 3A,B, right). D2-to-D3 co-differentiated myotubes presented significantly increased responses to KCl compared with those in the other groups on differentiation day 5 (Figure 3B, left), and the response of those myotubes to KCl was further increased on differentiation day 6 (Figure 3B, right). Compared with the control myotubes, the D2-to-D2, D3-to-D2, and D3-to-D3 co-differentiated myotubes did not show a significant change in their response to KCl at either tested time point (Figure 3A,B; Table 3).

The expression levels of four major proteins that mediate EC coupling were measured via immunoblot analysis using the lysates of D2-to-D3 co-differentiated myotubes obtained on differentiation day 5 (Figure 3C). Interestingly, the expression levels of RyR1 and SERCA1a (the primary isoform in adult fast-twitch skeletal muscle) were significantly increased, suggesting that increased expression of RyR1 and SERCA1a was responsible for the increased response to KCl in D2-to-D3 co-differentiated myotubes. In contrast, the expression levels of the triad formation-mediating proteins JP1 and JP2 were not changed, suggesting that the increased response to KCl in D2-to-D3 co-differentiated myotubes was not due to a change in the triad structure.

To examine the activity of RyR1 directly, co-differentiated myotubes were treated with caffeine (a direct agonist of RyR1). On differentiation day 5, the response of Mb-to-D2, D2-to-D2, and D3-to-D2 myotubes to caffeine was not significantly different from that of the control myotubes (Figure 4A, left). Similarly to the pattern of the response of intracellular Ca^2+^ to KCl, Mb-to-D2 and Mb-to-D3 co-differentiated myotubes on differentiation day 6 also presented significantly decreased responses to caffeine compared with those of the control myotubes (Figure 4A,B, right), suggesting that the decreased response to KCl is related to the decreased activity of RyR1. However, the significant increases in the caffeine response of D2-to-D3 co-differentiated myotubes on differentiation day 5 (Figure 4B, left) and the further increase in this increase on differentiation day 6 (Figure 4B, right) were consistent with the increased response to KCl and the increased expression of RyR1 in D2-to-D3 co-differentiated myotubes. These results suggest that the best appearance of D2-to-D3 co-differentiated myotubes is positively correlated with the functional increase in intracellular Ca^2+^ release.

### 3.4. D2-to-D3 Co-Differentiated Myotubes Showed Increases in the Intracellular Ca^2+^ Level and Extracellular Ca^2+^ Entry

**Figure 5:**
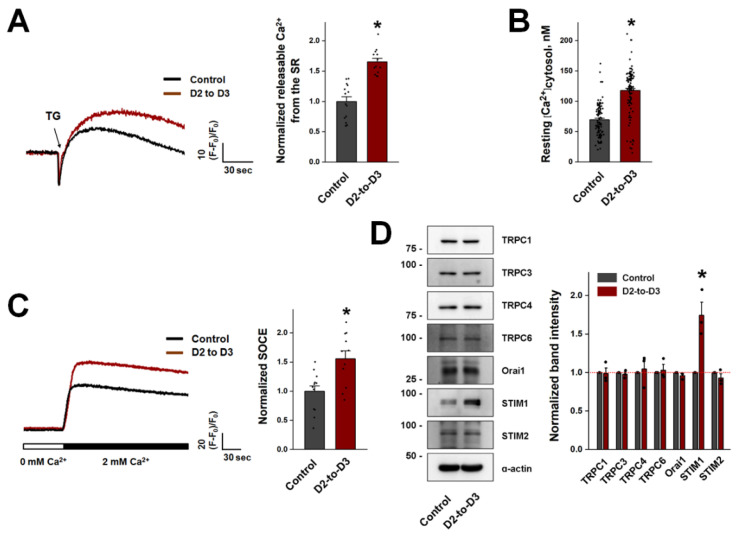
Amount of Ca^2+^ releasable from the SR, cytosolic Ca^2+^ level, SOCE, and expression level of SOCE-mediating proteins in D2-to-D3 co-differentiated myotubes. (**A**) The amount of Ca^2+^ releasable from the SR in D2-to-D3 co-differentiated myotubes on differentiation day 5 was measured after treatment with TG in the absence of extracellular Ca^2+^. (**B**) The resting cytosolic Ca^2+^ levels were measured, and the mean values are summarized as histograms. (**C**) SOCE was measured by depleting Ca^2+^ from the SR with TG in the absence of extracellular Ca^2+^ and then applying extracellular Ca^2+^ to the myotubes. A representative trace for each group is shown, and the results are summarized as histograms. The values were normalized to the mean values in the control group (**A**,**C**). The mean values are presented as the mean ± SE for the number of myotubes shown in parentheses in Table 5 (**A**–**C**). (**D**) Lysates of D2-to-D3 co-differentiated myotubes on differentiation day 5 were subjected to immunoblot analysis with antibodies against SOCE-mediating proteins. α-actin was used as the loading control. Three independent experiments per protein were conducted. A representative result is presented. The histogram shows the expression level of each protein normalized to the mean value in the control group (mean ± SE of three independent experiments, Table 6). * Significant difference compared with the control group (*p* < 0.05).

The intracellular Ca^2+^ level was examined in D2-to-D3 co-differentiated myotubes because Ca^2+^ is involved in the progression of terminal differentiation as well as in skeletal muscle contraction. First, the amount of Ca^2+^ releasable from the SR (i.e., the amount of Ca^2+^ in the SR) was measured by treatment with TG in the absence of extracellular Ca^2+^ to avoid possible entry of extracellular Ca^2+^ into the cytosol. The amount of Ca^2+^ releasable from the SR was significantly increased in D2-to-D3 co-differentiated myotubes compared with control myotubes (Figure 5A). In addition, the resting cytosolic Ca^2+^ level was significantly increased in D2-to-D3 co-differentiated myotubes (Figure 5B). To evaluate the possible contribution of extracellular Ca^2+^ entry to the increases in the amount of Ca^2+^ releasable from the SR and the cytosolic Ca^2+^ level, SOCE was measured in D2-to-D3 co-differentiated myotubes on differentiation day 5 (Figure 5C). SOCE was significantly increased in D2-to-D3 co-differentiated myotubes compared to control myotubes.

The expression levels of SOCE-mediating proteins in skeletal muscle were measured via immunoblot analysis using the lysates of D2-to-D3 co-differentiated myotubes (Figure 5D). STIM1 is a main protein that mediates SOCE in skeletal muscle, and its expression is significantly increased in D2-to-D3 co-differentiated myotubes, providing a possible explanation for the increased SOCE in D2-to-D3 co-differentiated myotubes.

## 4. Discussion

In this study, the transfer of myoblasts to host cells and their further co-differentiation into mature myotubes (Mb-to-D2 or Mb-to-D3 co-differentiated myotubes) did not result in an increased intracellular Ca^2+^ release compared with that in the control group (Figure 3 and Figure 4; Table 3 and Table 4), and the width of the co-differentiated myotubes on differentiation day 5 was significantly decreased (Table 1). A possible explanation for these findings is that myoblasts preferentially fuse with each other to form short and thin immature myotubes rather than fusing with relatively long and thick immature myotubes, such as the D2 or D3 immature myotubes used as the host cells. This preference could result in a significant decrease in the width of Mb-to-D2 and Mb-to-D3 co-differentiated myotubes on differentiation day 5, with further differentiation until differentiation day 6 required for recovery of the width (Table 1).

Compared with control myotubes, D2-to-D2 co-differentiated myotubes did not show a significance difference in width or intracellular Ca^2+^ release (Figure 3 and Figure 4; Table 1, Table 3 and Table 4), suggesting that neither the transfer of D2 immature myotubes nor the use of D2 immature myotubes as host cells affects their further differentiation. Unlike D2-to-D2 co-differentiated myotubes, D3-to-D3 co-differentiated myotubes showed a significant increase in width on differentiation day 5, but the width increase was temporary and was no longer detected on differentiation day 6 (Table 1). In addition, the increase in the width of myotubes on differentiation day 5 did not result in a functional increase in intracellular Ca^2+^ release in response to KCl (Figure 3B, left; and Table 3). Likewise, Mb-to-D2 co-differentiated myotubes on differentiation day 6 also showed no correlation between width and intracellular Ca^2+^ release (i.e., an increase in the width (Table 1) and a decrease in the intracellular Ca^2+^ release in response to KCl or caffeine compared with those on differentiation day 5 (Table 3 and Table 4)). These results suggest that improvements in myotube appearance (i.e., the formation of thicker myotubes or larger muscle bundles) are not always correlated with functional increases.

The increase in width of D2-to-D3 co-differentiated myotubes on differentiation day 5 could be the results of a synergy by the transferred D2 immature myotubes and the host D3 immature myotubes because neither the width of the host D3 nor the transferred D2 immature myotubes reached the width of D2-to-D3 co-differentiated myotubes (Figure S4). In addition, D2-to-D3 co-differentiated myotubes could differentiate faster than the control or other co-differentiated myotubes by reaching a significantly higher confluence on differentiation day 4 (Figure S5). Interestingly, unlike D2-to-D3 co-differentiated myotubes, D3-to-D2 co-differentiated myotubes did not exhibit improvements in appearance or function compared with control myotubes on differentiation day 5 or 6 (Table 1, Table 3 and Table 4). The most obvious difference between the D3-to-D2 and D2-to-D3 co-differentiation conditions was the large number of intersecting myotubes in the D3-to-D2 group on differentiation day 5 (Figure 2A, enlarged image 1, indicated by white arrowheads). These intersecting myotubes were also common in the D3-to-D3 group (Figure 2A, enlarged images 2 and 3, indicated by white arrowheads). There results suggest that co-differentiated myotubes acquire a different morphology when D3 immature myotubes are used as the transfer cells.

For accurate regulation of the cytosolic Ca^2+^ level in myotubes during skeletal muscle contraction and relaxation, timely and adequate expression of EC coupling- or SOCE-mediating proteins during terminal differentiation is needed. MyoD and myogenin are differentiation/myogenic factors that sequentially affect terminal differentiation [2]. In D2-to-D3 co-differentiated myotubes, the expression of MyoD but not myogenin was increased, and this increase could be positively related to the increased expression of major Ca^2+^ mobilization-mediating proteins (RyR1, SERCA1a, and STIM1; Figure 2C, Figure 3C and Figure 5D). Although the expression of both MyoD and myogenin is regulated by c-Myc [5,6], the mRNA expression of MyoD is increased as a compensatory mechanism for RyR1 silencing in C2C12 myotubes [12], and timely expression of myogenin during terminal differentiation could play a permissive role in the progression of terminal differentiation. It is possible that the greater expression of RyR1 and SERCA1a in D2-to-D3 co-differentiated myotubes could allow these myotubes to withstand the high Ca^2+^ levels in the SR and the cytosol (i.e., Ca^2+^ overload, as shown in Figure 5A,B) because more Ca^2+^ enters from the extracellular space through the SOCE mechanism, which could result in the smooth circulation of Ca^2+^ between the SR and the cytosol in D2-to-D3 co-differentiated myotubes. It seems that this possibility is reinforced by the further significant increase in the Ca^2+^ release of D2-to-D3 co-differentiated myotubes in response to caffeine on differentiation day 6 compared with day 5 (Figure 4B and Table 4). However, Mb-to-D2 or Mb-to-D3 co-differentiated myotubes that showed a significant decrease in Ca^2+^ release in response to KCl or caffeine did not show an increase in the expression level of MyoD (and a decrease in MyHC II expression correlated with the significant decrease in Ca^2+^ release in response to KCl or caffeine, Figure S6). Overall, MyoD could play an important role in the functional maturation of skeletal myotubes as well as in the progression of terminal differentiation.

Silencing STIM1 reduces SOCE and the terminal differentiation of human myoblasts to myotubes [21]. Likewise, knockdown of RyR1 by siRNA or inhibition of RyR1 activity reduces cytosolic Ca^2+^ level and blocks terminal differentiation [12]. An increase in the expression of myocyte enhancer factor-2 (MEF2, an early marker of terminal differentiation) and the activation of MEF2 via the Ca^2+^/calmodulin-dependent protein kinase pathway could be one of mechanisms by which the terminal differentiation of myoblasts into myotubes is accelerated by the increased cytosolic Ca^2+^ levels [21,22]. These previous studies suggest that the expression of STIM1 and RyR1 and the consequently enough cytosolic Ca^2+^ level play critical roles in terminal differentiation.

Overexpression of STIM1 with Orai1 increases SOCE and accelerates the terminal differentiation of human myoblasts to myotubes; however, overexpression of Orai1 alone does not affect SOCE [21]. This suggests that STIM1 is a limiting factor for SOCE activation even though Orai1 serves as the Ca^2+^ channel during SOCE. Therefore, it seems that the increased expression of only STIM1 is enough to increase SOCE in D2-to-D3 co-differentiated myotubes (with levels 1.6 times greater than in the control myotubes, as in Table 5). It is possible that SOCE in D2-to-D3 co-differentiated myotubes was increased by more than 1.6 times because, in addition to SERCA1a (i.e., Ca^2+^ uptake from the cytosol into the SR), immediate and parallel extrusion of the high cytosolic Ca^2+^ due to the high SOCE by plasma membrane Ca^2+^-ATPase and mitochondrial Ca^2+^ uptake by mitochondrial Ca^2+^ uniporter could exist [23,24]. On the other hand, STIM1 is expressed in myoblasts, and its expression is increased during terminal differentiation [20,25]. STIM1-mediated SOCE is necessary for neonatal muscle growth and differentiation because it maintains muscle fiber size via various Ca^2+^ signaling pathways [26]. In this study, the greater expression of STIM1 and the greater SOCE in D2-to-D3 co-differentiated myotubes is correlated with the increase in myotube width as well as the increase in Ca^2+^ in the SR and the cytosol, suggesting that during terminal differentiation of skeletal myoblasts into myotubes, the formation and function of myotubes are accomplished cooperatively via regulation of the available amount of Ca^2+^.

**Figure 6:**
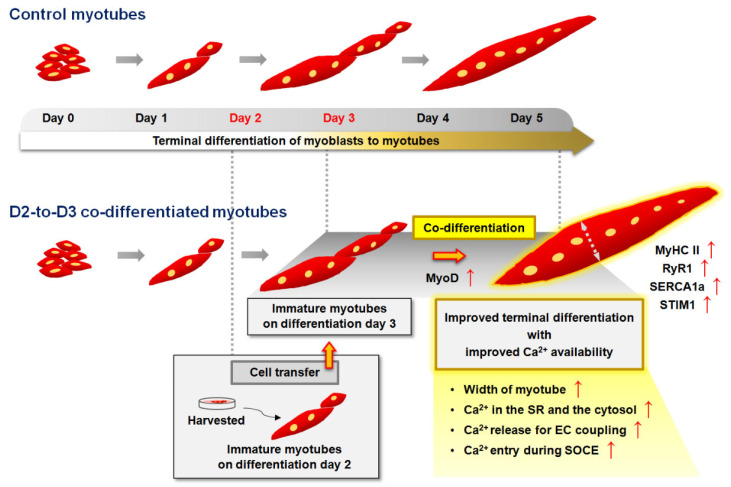
Summary schematic. Transfer of immature myotubes on differentiation day 2 to cultured immature myotubes on differentiation day 3 and the further co-differentiation of these myotubes into mature myotubes (termed D2-to-D3 co-differentiated myotubes in this study) effectively improved the appearance of myotubes and Ca^2+^ mobilization for skeletal muscle contraction. The width of the myotubes increased. The expression levels of MyoD (a myogenic factor), MyHC II (a contractile protein), RyR1 and SERCA1a (two major EC coupling-mediating proteins), and STIM1 (a SOCE-mediating protein) were increased, with the myotubes exhibiting a parallel and swirling alignment. The amounts of Ca^2+^ in the SR and the cytosol Ca^2+^ mobilization for skeletal muscle contraction (intracellular Ca^2+^ release for EC coupling and extracellular Ca^2+^ entry during SOCE) were increased.

In conclusion, co-differentiation with D2 immature skeletal myotubes as transfer cells and D3 immature skeletal myotubes as host cells improved myotube morphology and increased the amount of Ca^2+^ in the SR and the cytosolic Ca^2+^ mobilization for skeletal muscle contraction (as summarized in Figure 6). These findings are supported by the observed increases in the expression of MyoD, MyHC II, RyR1, SERCA1a, and STIM1, the myotube width, and the SR Ca^2+^ load. Taken together, the results of this study could provide insight into the possible use of in vitro-cultured mature myotubes that are co-differentiated from immature myotubes as a good alternative source of satellite cells or cultured myoblasts for the regeneration of injured or atrophied skeletal muscle.

## Figures and Tables

**Table 1:** Width of co-differentiated myotubes on differentiation day 5 or 6 (as shown in Figure 2A,B). The values were normalized to the mean values in the control group and are presented as the mean ± SE for 60 myotubes from 60 different spots in 5 wells per condition. The raw mean values of the control group on differentiation day 5 or 6 was 24.63 ± 0.74 or 25.20 ± 0.94 μm (mean ± S.E.), respectively. * Significant difference compared with the control group (*p* < 0.05). ^†^ Significant difference compared with the corresponding value on differentiation day 5 (*p* < 0.05).

	Co-Differentiation 1				Co-Differentiation 2			
	Control	Mb-to-D2	D2-to-D2	D3-to-D2	Control	Mb-to-D3	D2-to-D3	D3-to-D3
Differentiation day 5	1.00 ± 0.03	0.87 ± 0.03 *	0.93 ± 0.03	0.97 ± 0.04	1.00 ± 0.03	0.86 ± 0.03 *	1.35 ± 0.03 *	1.14 ± 0.03 *
Differentiation day 6	1.00 ± 0.03	1.08 ± 0.04 ^†^	0.99 ± 0.03	1.04 ± 0.05	1.00 ± 0.04	0.99 ± 0.03 ^†^	1.18 ± 0.04 *^,†^	1.09 ± 0.03

**Table 2:** Band intensities of myogenic factors, EC coupling-mediating proteins, and triad formation-mediating proteins in D2-to-D3 co-differentiated myotubes on differentiation day 5 (as shown in Figure 2C, Figure 3D and Figure 5D). The values were normalized to the mean values in the control group and are presented as the mean ± SE of three independent experiments per group. α-actin was used as a loading control. * Significant difference compared with the control (*p* < 0.05).

		Control	D2-to-D3
Myogenic factors	MyoD	1.00 ± 0.00	1.69 ± 0.20 *
	Myogenin	1.00 ± 0.00	1.02 ± 0.07
	MyHC II	1.00 ± 0.00	1.65 ± 0.23 *
α-actin for myogenic factors and MyHC II		1.00 ± 0.00	1.00 ± 0.02
EC coupling-mediating proteins	RyR1	1.00 ± 0.00	1.80 ± 0.37 *
	DHPR	1.00 ± 0.00	0.99 ± 0.08
	SERCA1a	1.00 ± 0.00	1.94 ± 0.37 *
	CASQ1	1.00 ± 0.00	0.98 ± 0.02
α-actin for EC coupling-mediating proteins		1.00 ± 0.00	1.02 ± 0.04
Triad formation-mediating proteins	junctophilin 1 (JP1)	1.00 ± 0.00	1.06 ± 0.15
	junctophilin 2 (JP2)	1.00 ± 0.00	0.96 ± 0.04
α-actin for triad formation-mediating proteins		1.00 ± 0.00	1.00 ± 0.04

**Table 3:** Intracellular Ca^2+^ release in response to KCl in co-differentiated myotubes on differentiation day 5 or 6 (as shown in Figure 3A,B). The values were normalized to the mean value in the control group and are presented as the mean ± SE for the number of myotubes shown in parentheses. * Significant difference compared with the control group (*p* < 0.05). ^†^ Significant difference compared with the corresponding value on differentiation day 5 (*p* < 0.05).

Co-Differentiation 1	Control	Mb-to-D2	D2-to-D2	D3-to-D2
Differentiation day 5	1.00 ± 0.13 (121 myotubes from 17 wells)	0.86 ± 0.11 (122 myotubes from 17 wells)	0.99 ± 0.12 (147 myotubes from 17 wells)	0.88 ± 0.07 (136 myotubes from 16 wells)
Differentiation day 6	1.00 ± 0.06 (147 myotubes from 19 wells)	0.52 ± 0.08 *^,†^ (70 myotubes from 14 wells)	0.99 ± 0.11 (93 myotubes from 13 wells)	0.99 ± 0.08 (77 myotubes from 13 wells)
**Co-Differentiation 2**	**Control**	**Mb-to-D3**	**D2-to-D3**	**D3-to-D3**
Differentiation day 5	1.00 ± 0.14 (109 myotubes from 14 wells)	0.94 ± 0.34 (74 myotubes from 14 wells)	3.15 ± 0.72 * (84 myotubes from 12 wells)	1.13 ± 0.23 (75 myotubes from 12 wells)
Differentiation day 6	1.00 ± 0.13 (92 myotubes from 13 wells)	0.58 ± 0.15 * (78 myotubes from 11 wells)	4.44 ± 0.80 * (93 myotubes from 12 wells)	1.75 ± 0.60 (77 myotubes from 12 wells)

**Table 4:** Intracellular Ca^2+^ release in response to caffeine in co-differentiated myotubes on differentiation day 5 or 6 (as shown in Figure 4). The values were normalized to the mean value in the control group and are presented as the mean ± SE for the number of myotubes shown in parentheses. * Significant difference compared with the control group (*p* < 0.05). ^†^ Significant difference compared with the corresponding value on differentiation day 5 (*p* < 0.05).

Co-Differentiation 1	Control	Mb-to-D2	D2-to-D2	D3-to-D2
Differentiation day 5	1.00 ± 0.11 (146 myotubes from 19 wells)	1.10 ± 0.08 (156 myotubes from 19 wells)	1.08 ± 0.15 (151 myotubes from 19 wells)	1.02 ± 0.11 (127 myotubes from 19 wells)
Differentiation day 6	1.00 ± 0.08 (151 myotubes from 20 wells)	0.74 ± 0.09 *^,†^ (80 myotubes from 16 wells)	0.94 ± 0.12 (114 myotubes from 16 wells)	0.98 ± 0.10 (93 myotubes from 16 wells)
**Co-Differentiation 2**	**Control**	**Mb-to-D3**	**D2-to-D3**	**D3-to-D3**
Differentiation day 5	1.00 ± 0.10 (146 myotubes from 19 wells)	0.95 ± 0.12 (156 myotubes from 19 wells)	1.92 ± 0.27 * (151 myotubes from 19 wells)	1.19 ± 0.16 (127 myotubes from 19 wells)
Differentiation day 6	1.00 ± 0.09 (105 myotubes from 15 wells)	0.70 ± 0.18 * (90 myotubes from 13 wells)	3.10 ± 0.59 *^,†^ (101 myotubes from 15 wells)	1.60 ± 0.40 (86 myotubes from 13 wells)

**Table 5:** Ca^2+^ level in the SR, cytosolic Ca^2+^ level, and SOCE in D2-to-D3 co-differentiated myotubes on differentiation day 5 (as shown in Figure 5A–C). The values are presented as the mean ± SE for the number of myotubes shown in parentheses. For releasable Ca^2+^ from the SR and SOCE, the values were normalized to the mean values in the control group. * Significant difference compared with the control group (*p* < 0.05).

	Control	D2-to-D3
Releasable Ca^2+^ from the SR	1.00 ± 0.08 (94 myotubes from 14 wells)	1.65 ± 0.06 * (98 myotubes from 14 wells)
Cytosolic [Ca^2+^], nM	69.38 ± 2.58 (104 myotubes from 16 wells)	117.89 ± 3.71 * (106 myotubes from 16 wells)
SOCE	1.00 ± 0.09 (85 myotubes from 14 wells)	1.56 ± 0.14 * (86 myotubes from 14 wells)

**Table 6:** Band intensities of SOCE-mediating proteins in D2-to-D3 co-differentiated myotubes at differentiation day 5 (as shown in Figure 5D). The values were normalized to the mean values in the control group and are presented as the mean ± SE of three independent experiments per protein. α-actin was used as the loading control. * Significant difference compared with the control group (*p* < 0.05).

	Control	D2-to-D3
TRPC1	1.00 ± 0.00	0.99 ± 0.08
TRPC3	1.00 ± 0.00	0.98 ± 0.03
TRPC4	1.00 ± 0.00	1.05 ± 0.12
TRPC6	1.00 ± 0.00	1.03 ± 0.08
Orai1	1.00 ± 0.00	0.96 ± 0.02
STIM1	1.00 ± 0.00	1.74 ± 0.17 *
STIM2	1.00 ± 0.00	0.93 ± 0.05
α-actin	1.00 ± 0.00	1.00 ± 0.05

## Data Availability

The original contributions presented in the study are included in the article/Supplementary Materials; further inquiries can be directed to the corresponding author.

## Supplementary Materials

The following supporting information can be downloaded at: https://www.mdpi.com/article/10.3390/cells13242136/s1. Figure S1: Attachment rate, Figure S2: The original file for the main figure in Figure 2A, Figure S3: Fusion index, Figure S4: Myotube width in different conditions, Figure S5: Confluence, Figure S6: Expression level of MyoD or MyHC II in Mb-to-D2 or Mb-to-D3 co-differentiated myotubes, Table S1: Definitions of the different types of muscle cells mentioned in this study, Table S2: Numbers of myoblasts on differentiation day 0 under the six different co-differentiation conditions, Table S3: Numbers of spots observed for counting myoblasts on differentiation day 0), Table S4: Antibodies, Table S5: Number of observed spots for width measurement.
