# Supplementary material for: Immature Skeletal Myotubes Are an Effective Source for Improving the Terminal Differentiation of Skeletal Muscle
Source: Cells. 2024 Dec 23;13(24):2136. doi: 10.3390/cells13242136 (PMC11674136; doi:10.3390/cells13242136)
Supplement: Supplementary file 1 [file cells-13-02136-s001.zip › cells-3322777-supplementary.pdf]

Figure S1. Attachment rate. Myoblasts, D2 immature myotubes, or D3 immature myotubes were transferred to empty plates that were coated with Matrigel, and the differentiation condition were applied to them. The number of cells on the plates were counted by staining nuclei with DAPI at 1h, 4h, 7h after the transfer. The number of cells without transfer at the same time for each cell type were regarded as 100%. Myoblasts showed attachment rate of 48.33 ± 4.56 % at 1h after the transfer in a unit area (1,250 μm wide and 950 μm long), which was not significantly changed over the time (4h or 7h after the transfer). The data are summarized in histograms. The mean values are presented as the mean ± SE for five independent areas per each. The same tendency of attachment rate was found with D2 or D3 immature myotubes.

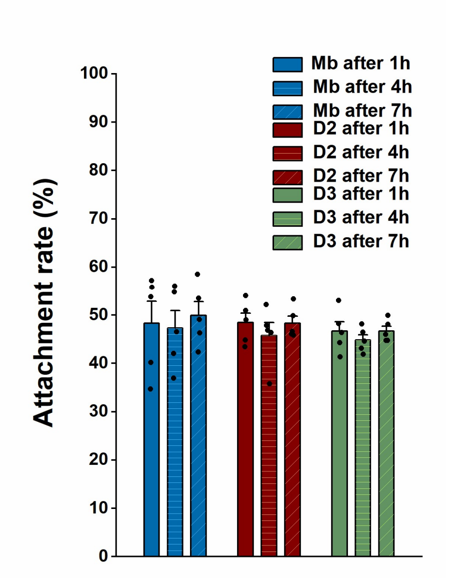

		Attachment rate (%)
Mb	After 1h	48.33 ± 4.56
	After 4h	47.26 ± 3.66
	After 7h	49.94 ± 2.81
D2	After 1h	48.45 ± 1.96
	After 4h	45.78 ± 2.71
	After 7h	48.31 ± 1.44
D3	After 1h	46.64 ± 1.95
	After 4h	44.81 ± 1.12
	After 7h	46.68 ± 1.02
Significant difference compared with Mb after 1h (P < 0.05), however there was no significant difference.		

Figure S2. The original file for the main figure in Figure 2A.

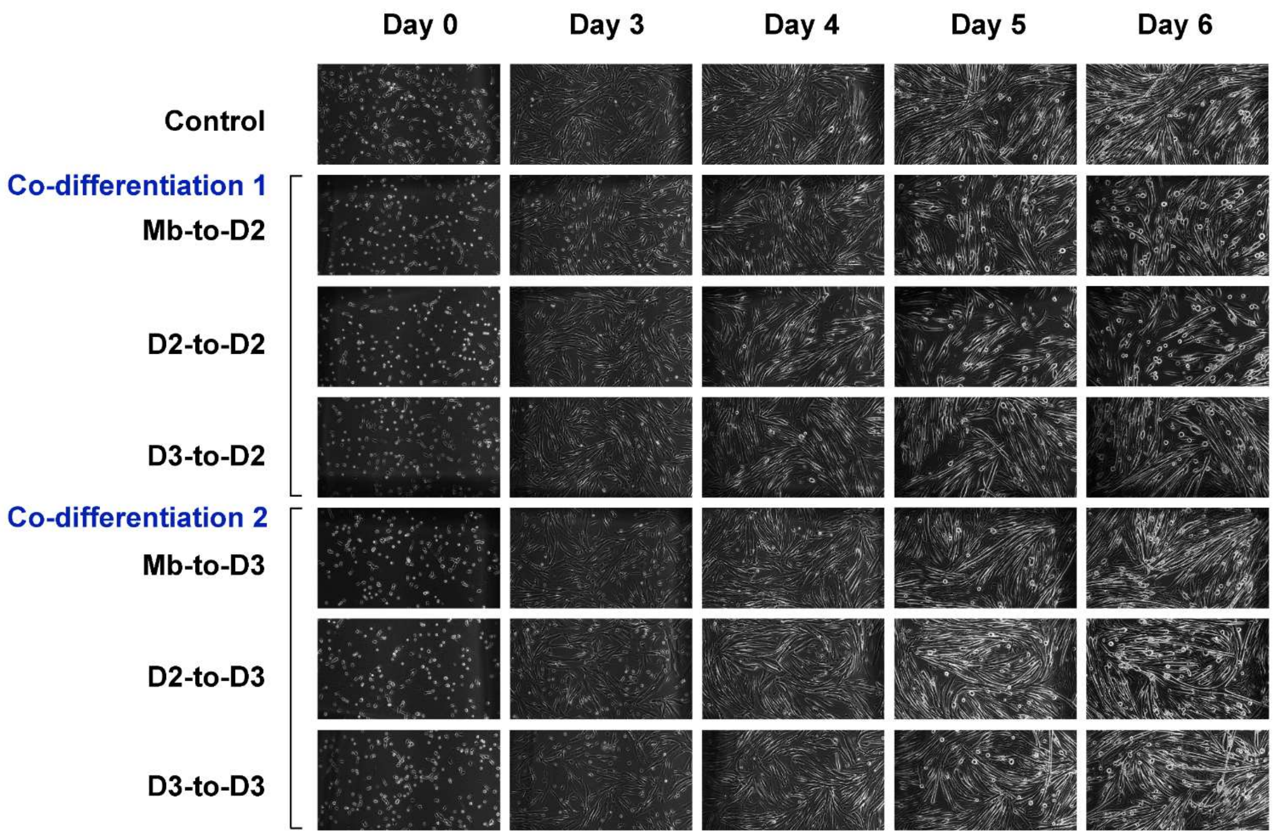

Figure S3. Fusion index. Fusion indexes on differentiation day 5 of control myotubes without cell transfer (Control), D2 immature myotubes (D2), D3 immature myotubes (D3), or D2-to-D3 codifferentiated myotubes (D2-to-D3) were obtained by staining cells in a unit area (1,250 µm wide and 950 µm long) with DAPI and using the Figi imaging program (Schindelin J, Arganda-Carreras I, Frise E, Kaynig V, Longair M, Pietzsch T, Preibisch S, Rueden C, Saalfeld S, Schmid B, Tinevez JY, White DJ, Hartenstein V, Eliceiri K, Tomancak P, Cardona A. Fiji: an open-source platform for biological-image analysis. Nat Methods. 2012 Jun 28;9(7):676-82. doi: 10.1038/nmeth.2019). The data are summarized in histograms (mean ±SE for four independent areas).

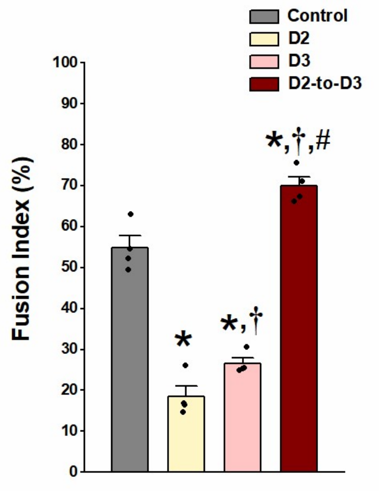

	Fusion index (%)
Control myotubes on differentiation day 5 (Control)	54.83 ± 2.90
D2 immature myotubes (D2)	18.48 ± 2.55 *
D3 immature myotubes (D3)	26.58± 1.32 *,†
D2-to-D3 codifferenitated myotubes on differentiation day 5 (D2-to-D3)	70.00 ± 2.11 *,†,#

* Significant difference compared with the Control ($P < 0.05$).
† Significant difference compared with the D2 ($P < 0.05$).
# Significant difference compared with the D3 ($P < 0.05$).
Significant differences ($P < 0.05$) were identified via one-way ANOVA with Tukey's post hoc test.

Figure S4. Myotube width in different conditions. D2 immature myotubes were transferred to host D3 immature myotubes, and they were further codifferentiated for 2 days (D2-to-D3); host D3 immature myotubes without cell transfer were further differentiated for 2 days (host D3); D2 immature myotubes without host cells were further differentiated for 2 days (D2); D2 immature myotubes were transferred to empty dishes coated with Matrigel and further differentiated for 2 days (Transferred D2). The numbers of myoblasts in Table S2 were used for preparing the transferred cells or host cells. In case of 'Transferred D2', twice number of myoblasts was used because the attachment rate of D2 immature myotubes after cell transfer was approximately 50% (48.45 ± 1.96 %, Figure S1). The data are summarized in histograms (mean ± SE for forty independent myotubes). Neither the width of D3 (host D3) nor D2 cells (D2) allowed to differentiate alone reached the width of D2-to-D3, suggesting that the increase in the width of D2-to-D3 codifferentiated myotubes in Figure 2B could be the results of a synergy by the transferred D2 immature myotubes and host D3 immature myotubes. In addition, widths of 'Transferred D2' were not significantly different from those of D2, suggesting that a transfer of D2 immature myotubes from one to another plate does not affect the width of myotubes.

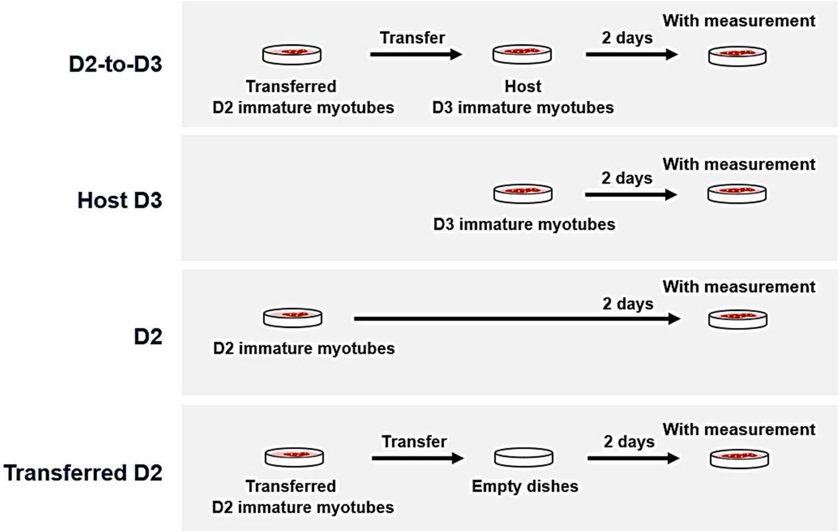

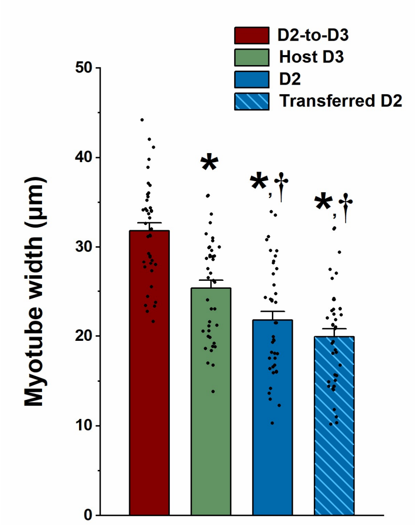

	Myotube width (μm)
D2-to-D3	31.83 ± 0.88
Host D3	25.34 ± 0.90 *
D2	21.86 ± 0.99 *,†
Transferred D2	19.95 ± 0.89 *,†

* Significant difference compared with D2-to-D3 (*P* < 0.05)
† Significant difference compared with Host D3 (*P* < 0.05)
Significant differences (*P* < 0.05) were identified via one-way ANOVA with Tukey's post hoc test.

Figure S5. Confluence. The number of myoblasts in Table S2 was used for preparing the transferred cells or host cells. Confluence of D3 host cells on differentiation day 3 were 65.77 ± 1.08 %. At 24 hrs (i.e., differentiation day 4) after transferring myoblasts, D2 immature myotubes or D3 immature myotubes to the D3 host cells, confluence of codifferentiated myotubes was measured in a unit area (1,250 μm wide and 950 μm long) using the Figi imaging program. The data are summarized in histograms (mean ± SE for eight independent experiments). The white bar in the bottom 279 right-hand corner of the main figure represents 100 μm. Confluence of D2-to-D3 codifferentiated myotubes was significantly increased 87.55 ± 0.99 % compared with the control myotube without cell transfer, but this difference was not found in Mb-to-D3 or D3-to-D3 codifferentiated myotubes. This result suggests that, considering that confluence of codifferentiated myotubes as well as control myotubes on differentiation day 5 is approximately 90%, D2-to-D3 codifferentiated myotubes differentiates faster than other types of codifferentiated myotubes.

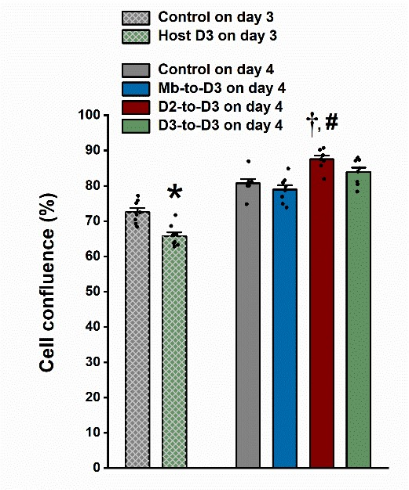

	Cell confluence (%)
Control on day 3	72.55 ± 1.14
Host D3 on day 3	65.77 ± 1.08 *
* Significant difference compared with the Control on day 3 ($P < 0.05$). Significant differences ($P < 0.05$) were identified via unpaired t test.	
Control on day 4	80.73 ± 1.16
Mb-to-D3 on day 4	78.93 ± 1.29
D2-to-D3 on day 4	87.55 ± 0.99 †,#
D3-to-D3 on day 4	83.91 ± 1.27
† Significant difference compared with Control on day 4 ($P < 0.05$); # Significant difference compared with Mb-to-D3 on day 4 ($P < 0.05$). Significant differences ($P < 0.05$) were identified via one-way ANOVA with Tukey's post hoc test.	

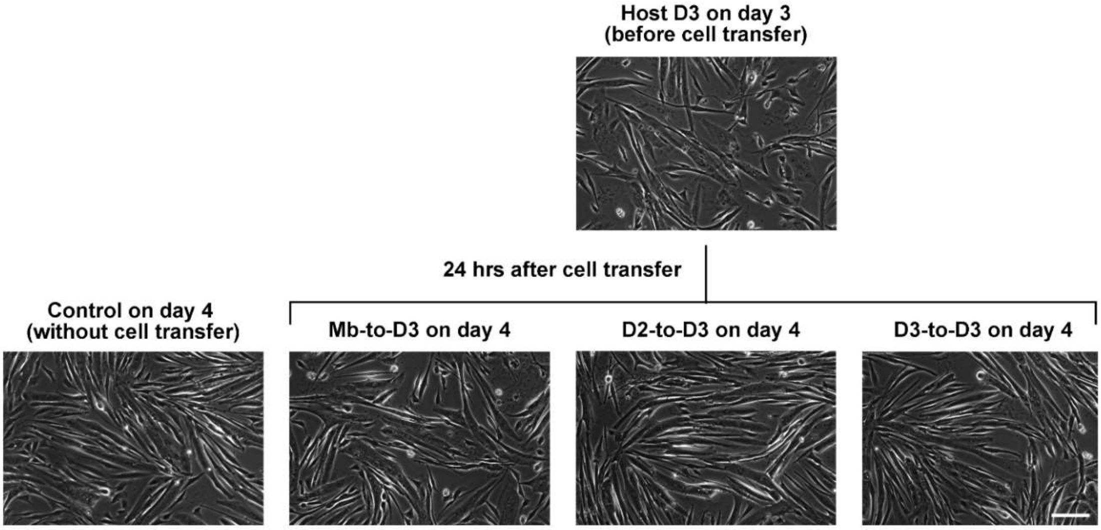

Figure S6. Expression level of MyoD or MyHC II in Mb-to-D2 or Mb-to-D3 co-differentiated myotubes. Lysates of Mb-to-D2 or Mb-to-D3 co-differentiated myotubes on differentiation day 5 were subjected to immunoblot analysis with antibodies against MyoD and MyHC II. α-actin was used as the loading control. A representative result is presented. The expression level of each protein was normalized to the mean value in the control group, and the data are summarized in histograms (mean ± SE of three independent experiments). *Significant difference compared with the control group ($P < 0.05$).

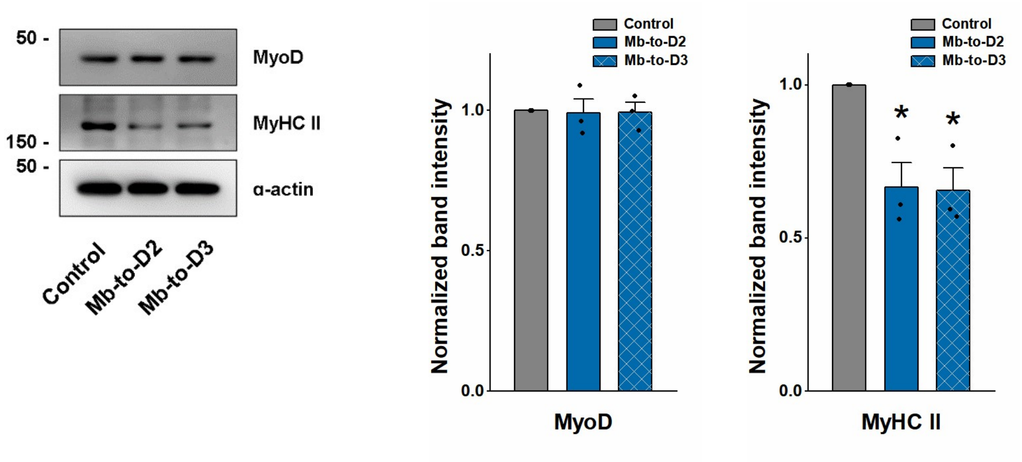

		Control	Mb-to-D2	Mb-to-D3
Normalized band intensity	MyoD	1.00 ± 0.00	0.99 ± 0.05	0.99 ± 0.04
	MyHC II	1.00 ± 0.00	0.67 ± 0.08 *	0.65 ± 0.07 *
* Significant difference compared with D2-to-D3 ($P < 0.05$)				

Table S1. Definitions of the different types of muscle cells mentioned in this study.

Name	Definition
**Satellite cells** (also called 'myosatellite cells' or 'muscle stem cells' in recent articles)	- Precursor cells to skeletal muscle cells<br>- Quiescent satellite cells: they neither proliferate nor differentiate.<br>- Activated satellite cells: they can proliferate, and the proliferated ones (i.e., myoblasts) can differentiate to myotubes
**Myoblasts**	- Cells derived from the activated satellite cells<br>- They can proliferate and differentiate to myotubes.
**Myotubes**	- Multinucleated and differentiated cells by the fusion of myoblasts
**Muscle fibers** (also called 'myofibers')	- Mature multinucleated cells *in vivo*

Table S2. Numbers of myoblasts on differentiation day 0 under the six different codifferentiation conditions. The mean values are presented as the mean ± SE for the indicated number of myoblasts. Significant difference compared with the Mb-to-D2 group for codifferentiation 1 or the Mb-to-D3 group for codifferentiation 2 ($P < 0.05$), however there was no significant difference.

Experimental condition		Number of myoblasts in a unit are (1,000 µm wide and 600 µm long) on differentiation day 0	
		Transferred cells	D2 or D3 host cells
Codifferentiation 1 (D2 host cells)	Control	None	137.53 ± 19.96
	Mb-to-D2	111.80 ± 22.93	112.30 ± 19.94
	D2-to-D2	99.28 ± 17.90	102.73 ± 18.41
	D3-to-D2	114.26 ± 20.11	108.26 ± 20.90
Codifferentiation 2 (D3 host cells)	Control	None	144.49 ± 17.12
	Mb-to-D3	117.59 ± 19.33	101.90 ± 18.75
	D2-to-D3	92.64 ± 15.96	93.87 ± 15.56
	D3-to-D3	103.08 ± 18.21	104.33 ± 17.76

Table S3. Numbers of spots observed for counting myoblasts on differentiation day 0. Spots were randomly selected from a unit area (1,000 µm wide and 600 µm long) in different wells.

Experimental condition		Number of observed spots
Codifferentiation 1	Control	120 spots in 10 wells
	Mb-to-D2	192 spots in 16 wells
	D2-to-D2	240 spots in 20 wells
	D3-to-D2	192 spots in 16 wells
Codifferentiation 2	Control	120 spots in 10 wells
	Mb-to-D3	240 spots in 20 wells
	D2-to-D3	288 spots in 24 wells
	D3-to-D3	240 spots in 20 wells

Table S4. Antibodies. The antibodies were diluted 1:1,000 for use. The secondary antibodies were diluted 1:50,000.

Name of antibody	Company	Catalog #
Anti-RyR1 antibody	Thermo Fisher Scientific (Waltham, MA, USA)	MA3-925
Anti-DHPR antibody		MA3-920
Anti-SERCA1a antibody		MA3-912
Anti-CASQ1 antibody		MA3-913
Anti-JP1 antibody		40-5100
Anti-TRPC1 antibody	Alomone Labs (Jerusalem, Israel)	ACC-010
Anti-TRPC3 antibody		ACC-016
Anti-TRPC4 antibody		ACC-018
Anti-TRPC6 antibody		ACC-017
Anti-Orai1 antibody	Santa Cruz Biotechnology (Dallas, TX, USA)	sc-377281
Anti-α-actin antibody		sc-58671
Anti-MyoD antibody		sc-377460
Anti-myogenin antibody		sc-398002
Anti-JP2 antibody		sc-134875
Anti-MyHC II antibody	Abcam (Cambridge, MA, USA)	ab37484
Anti-STIM1 antibody	Cell Signaling Technology (Danvers, MA, USA)	4916
Anti-STIM2 antibody	Proteintech (Rosemont, IL, USA)	21192-1-AP
Anti-goat secondary antibody	Jackson Immuno Research Laboratories	205-035-108
Anti-mouse secondary antibody	(West Grove, PA, USA)	715-035-151
Anti-rabbit secondary antibody		711035-152

Table S5. Number of observed spots for width measurement (in Figure 2B and Table 1). Spots were randomly selected from a unit area (1,250 µm wide and 950 µm long) in different wells.

Experimental condition		Number of observed spots
Codifferentiation 1	Control	60 spots in 5 wells
	Mb-to-D2	60 spots in 5 wells
	D2-to-D2	60 spots in 5 wells
	D3-to-D2	60 spots in 5 wells
Codifferentiation 2	Control	60 spots in 5 wells
	Mb-to-D3	60 spots in 5 wells
	D2-to-D3	60 spots in 5 wells
	D3-to-D3	60 spots in 5 wells
